# Report of South Dakota State Dental Association

**Published:** 1904-02

**Authors:** 


### REPORT OF SOUTH DAKOTA STATE DENTAL ASSOCIATION.

The 21st annual meeting of the South Dakota State Dental Association was held at Redfield, S. D., June 3, 4 and 5.

The meeting was under the direction of Dr. J. P. Buckley of Chicago, who, by means of lectures and clinics, worked along the line of oral therapeutics.

Dr. A. O. Hunt, of Omaha, was present and gave a talk on porcelain, calling attention to the use of Brewster's oil colors for staining porcelain teeth to imitate nature.

The following officers were elected for the ensuing year: Dr. C. E. Stutenroth, Redfield, Pres.; Dr. E. S. O'Neal, Canton, V. P.; Dr. J. W. Ross, Milbank, Sec'y; Dr. W. H. Jackson, Flandreau, Treas., Dr. D. W. I. Davies, Librarian.

The next meeting will be held at Aberdeen, June, 1904. While the society is small, its members are enthusiastic and progressive men, and are doing much to advance the standing of our profession in the northwest.

Pres. Davies, in his annual address, paid a glowing tribute to the late Dr. F. W. Blomily, of Sioux Falls.

